# Comorbid Chronic Diseases and Acute Organ Injuries Are Strongly Correlated with Disease Severity and Mortality among COVID-19 Patients: A Systemic Review and Meta-Analysis

**DOI:** 10.34133/2020/2402961

**Published:** 2020-04-19

**Authors:** Xinhui Wang, Xuexian Fang, Zhaoxian Cai, Xiaotian Wu, Xiaotong Gao, Junxia Min, Fudi Wang

**Affiliations:** The First Affiliated Hospital, School of Public Health, Institute of Translational Medicine, Zhejiang University School of Medicine, Hangzhou 310058, China

## Abstract

The recent outbreak of COVID-19 has been rapidly spreading on a global scale. To date, there is no specific vaccine against the causative virus, SARS-CoV-2, nor is there an effective medicine for treating COVID-19, thus raising concerns with respect to the effect of risk factors such as clinical course and pathophysiological parameters on disease severity and outcome in patients with COVID-19. By extracting and analyzing all available published clinical data, we identified several major clinical characteristics associated with increased disease severity and mortality among patients with COVID-19. Specifically, preexisting chronic conditions such as hypertension, cardiovascular disease, chronic kidney disease, and diabetes are strongly associated with an increased risk of developing severe COVID-19; surprisingly, however, we found no correlation between chronic liver disease and increased disease severity. In addition, we found that both acute cardiac injury and acute kidney injury are highly correlated with an increased risk of COVID-19-related mortality. Given the high risk of comorbidity and the high mortality rate associated with tissue damage, organ function should be monitored closely in patients diagnosed with COVID-19, and this approach should be included when establishing new guidelines for managing these high-risk patients. Moreover, additional clinical data are needed in order to determine whether a supportive therapy can help mitigate the development of severe, potentially fatal complications, and further studies are needed to identify the pathophysiology and the mechanism underlying this novel coronavirus-associated infectious disease. Taken together, these findings provide new insights regarding clinical strategies for improving the management and outcome of patients with COVID-19.

## 1. Introduction

**Figure 1:**
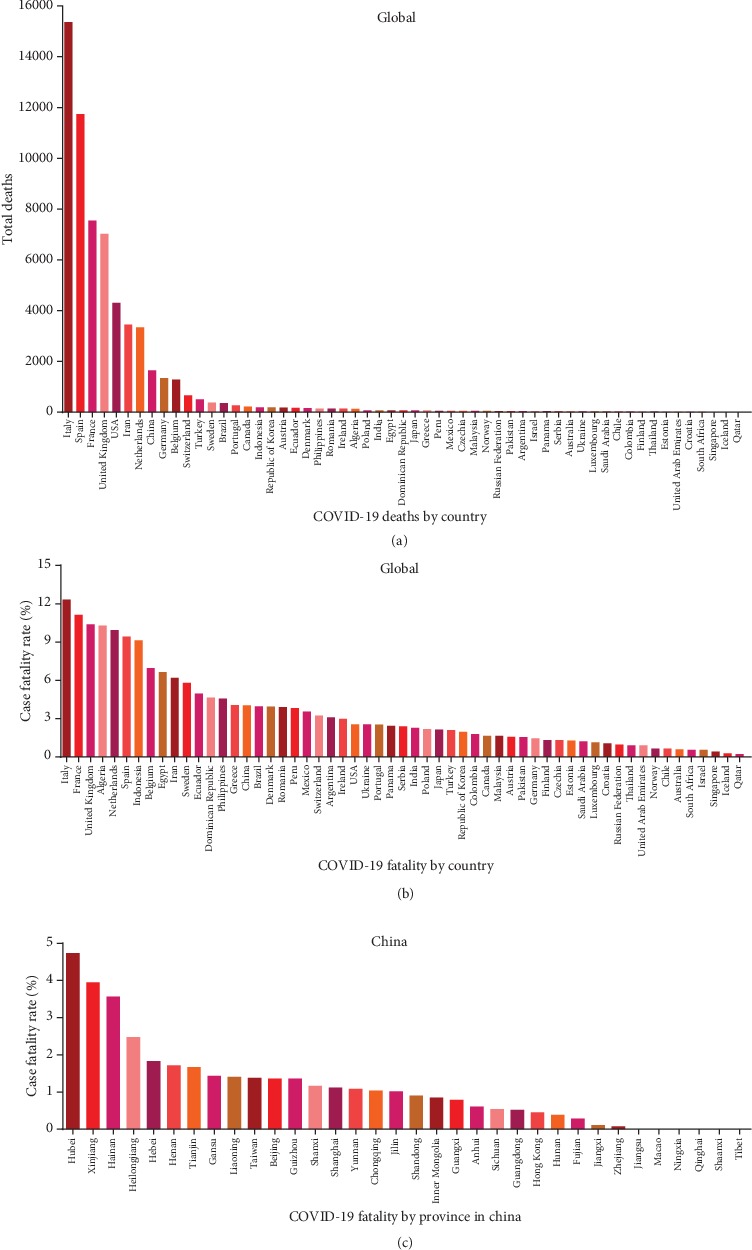
Summary of the total number of deaths and mortality rate among SARS-CoV-2-infected patients recorded through April 6, 2020. (a, b) Summary of the total number of deaths (a) and mortality rate (b) in the indicated countries with more than 1,000 total cases reported; data were retrieved from the World Health Organization. (c) Summary of the mortality rate in the indicated regions in China (including Hong Kong, Macao, and Taiwan); data were retrieved from the Chinese Center for Disease Control and Prevention.

The recently identified novel SARS-CoV-2 virus has caused an outbreak of the underlying disease, COVID-19, which has continued to spread rapidly throughout China and around the world. As of April 6, 2020, a total of 1,174,866 COVID-19 cases and 64,541-related deaths were reported in 209 countries, areas, or territories spanning six continents, with 83,071 cases and 3,340 deaths reported in China alone. There is currently no effective vaccine or antiviral medication available for SARS-CoV-2. In addition, the case-fatality (i.e., COVID-19-related mortality) rate varies widely among epicenters and counties, even at the global level (Figure 1). To reduce the overall mortality rate, identifying risk factors associated with disease severity and poor outcome among COVID-19 patients is urgently needed. Therefore, COVID-19 patients who present with a comorbid condition may have an increased risk of deterioration and should therefore be admitted to a designated unit for close monitoring in accordance with the WHO guidelines for screening and triage [1]. Importantly, the ability to accurately evaluate risk factors associated with poor prognosis among SARS-CoV-2-infected patients is essential for early intervention in order to improve these patients' prognosis. At the same time, identifying patients who are at risk of developing severe disease could help healthcare providers allocate their limited care resources more effectively in SARS-CoV-2-infected communities.

Previous retrospective studies reported an increased risk of developing more severe complications in COVID-19 patients with certain preexisting chronic diseases [2–4]. In addition, the development of acute organ damage and/or dysfunction has also been linked to increased severity and higher mortality rates among COVID-19 patients [2, 5–16]. However, to date, no systematic review or meta-analysis has been reported regarding the putative association between various risk factors and prognosis in COVID-19 patients, with the sole exception of acute respiratory distress syndrome (ARDS).

**Figure 2:**
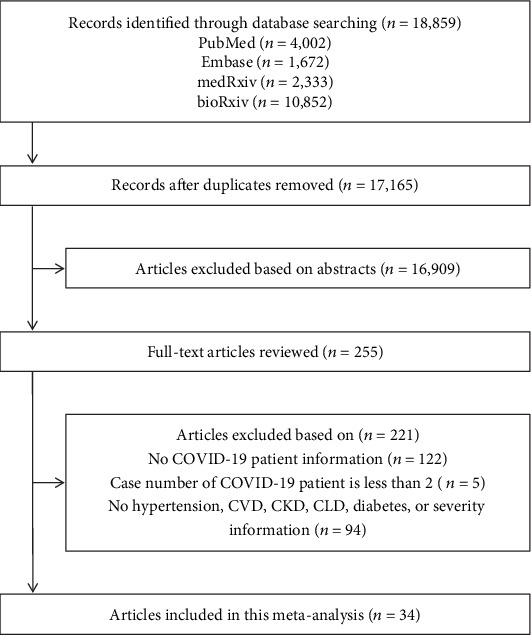
Flow-chart depicting the literature search and selection strategy. After applying the inclusion and exclusion criteria, a total of 34 articles were included in the final meta-analysis.

Here, we performed a systematic review and meta-analysis in order to identify risk factors associated with the severity and mortality rate among COVID-19 patients. We searched the PubMed, Embase, Web of Science, medRxiv, and bioRxiv databases for articles published through April 6, 2020. After removing duplicate publications, excluding articles based on the abstract, and screening the remaining articles by reading the full-text publication, a total of 34 studies were included in our final analysis (Figure 2), with a total of 6,263 COVID-19 cases, including 1,727 and 4,536 severe and nonsevere patients, respectively (Table 1). We then extracted data regarding the outcomes of interest from the studies, and the pooled results were analyzed using a random-effects model. Specifically, we analyzed the effect of various preexisting chronic diseases on the risk of developing severe COVID-19, as well as the clinical characteristics of organ injury in patients with severe COVID-19.

## 2. Results

### 2.1. Cardiac Comorbidity and Acute Heart Injury Are Associated with Increased Disease Severity in Patients with COVID-19

**Figure 3:**
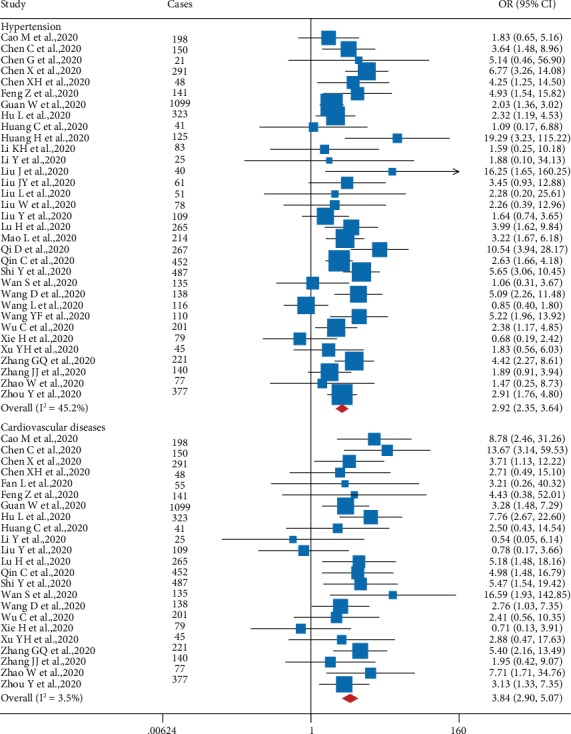
Forest plot showing the effect of comorbid hypertension (top) and cardiovascular disease (bottom) on the risk of severe COVID-19 in SARS-CoV-2-infected patients. In this and subsequent figures, the horizontal lines indicate the lower and upper limits of the 95% CI, and the size of the blue squares reflects the relative weight of each study in the meta-analysis. OR: odds ratio.

The mechanisms that underlie the development of severe COVID-19 are poorly understood and warrant further investigation. Huang et al. [5] and Wang et al. [6] previously suggested that preexisting heart disease could be a potential risk factor for SARS-CoV-2-infected patients being admitted to the ICU. To test this, we performed a meta-analysis in order to investigate whether cardiovascular disease (CVD) and/or hypertension is significantly associated with increased disease severity in SARS-CoV-2-infected patients. Our analysis revealed that compared to COVID-19 patients with no preexisting chronic cardiovascular condition, COVID-19 patients who present with either hypertension or CVD have an approximately 3-4-fold higher risk of developing severe disease, with an odds ratio (OR) of 2.92 (95% CI: 2.35, 3.64) and 3.84 (95% CI: 2.90, 5.07), respectively (Figure 3). In addition, our analysis revealed moderate and low heterogeneity among the included studies with respect to hypertension (*I*^2^ = 45.2%) and CVD (*I*^2^ = 3.5%). Based on these results, we suggest that COVID-19 patients who present with a history of hypertension and/or heart disease should be carefully monitored and managed.

During the progression of COVID-19, complications such as acute cardiac injury (ACI) can occur due to an unknown mechanism, particularly among severe cases. We therefore systematically examined the correlation between ACI and COVID-19 severity. The epidemiological characteristics of cardiac injury in COVID-19 patients were extracted and are summarized in Table 2. The first report of ACI in patients infected with SARS-CoV-2 was a retrospective study by Huang et al. based on a report from Jinyintan Hospital in Wuhan, China [5], which included 41 laboratory-confirmed COVID-19 cases; five of these 41 patients (12%) had ACI, and four of these five patients (80%) were admitted to the ICU. In addition, Wang et al. studied an additional 138 COVID-19 patients in Wuhan, China, and found that 10 patients (7.2%) were diagnosed with virus-related ACI [6]. Wang et al. also found that COVID-19 patients admitted to the ICU were more likely to have cardiac complications (22.2%) compared to patients who were not admitted to the ICU (2.0%) [6]. Zhang et al. reported that 29.1% of severe COVID-19 patients in Zhongnan Hospital at Wuhan University had ACI [10]. Yang et al. treated 52 critically ill adults with SARS-CoV-2 infection in the ICU at Jinyintan Hospital, 32 of whom (61.5%) died during treatment [9]. They found that 12 of the 52 patients (23%) had myocardial injury, indicating that patients with this condition have a higher risk of death; moreover, a closer analysis revealed that nonsurviving patients had a nearly 2-fold higher risk of developing ACI compared to surviving patients [9]. Recently, a relatively large epidemiology survey found a strong association between ACI and COVID-19-related mortality [12].

Investigators in Beijing measured serum troponin I (TnI) levels in patients with light, mild, severe, and critical COVID-19 and found that this sensitive marker for ACI was elevated in all critical patients [7]; in addition, computed tomography (CT) scans revealed a low density of epicardial adipose tissue, indicating increased cardiac inflammation, in severe and critical patients. Xu et al. found that intubated COVID-19 patients had a much higher risk of developing ACI compared to nonintubated patients in the ICU [11]. Wu et al. also analyzed ACI-related markers, including TnI, creatine kinase-MB, lactate dehydrogenase (LDH), and *α*-hydroxybutyrate dehydrogenase, and found that COVID-19 patients who were admitted with increased serum levels of these markers had significantly higher overall mortality rates and shorter survival [8]. Thus, COVID-19 patients who develop signs of ACI should be identified as early as possible, and cardiovascular specialists should be consulted in order to minimize the risk of heart damage-related mortality.

### 2.2. Chronic Kidney Disease and Acute Kidney Injury Are Strongly Correlated with Increased Disease Severity in COVID-19 Patients

**Figure 4:**
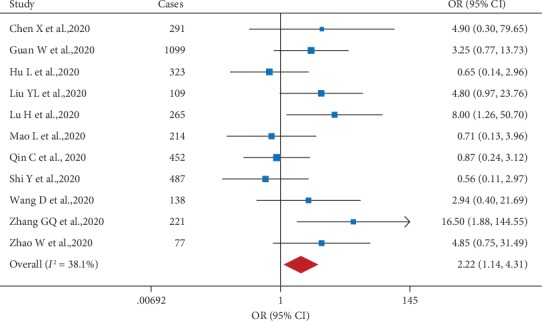
Forest plot showing the effect of comorbid chronic kidney disease on the risk of severe COVID-19 in SARS-CoV-2-infected patients.

Next, we performed a meta-analysis in order to examine the association between preexisting chronic kidney disease (CKD) and disease severity in patients with COVID-19. We found that CKD was strongly correlated with increased disease severity (OR: 2.22; 95% CI: 1.14, 4.31), with moderate heterogeneity (*I*^2^ = 38.1%) (Figure 4). It is interesting to note that patients with CKD often present with anemia, hypertension, and/or cardiovascular disease [17–19]; in this respect, we suggest that COVID-19 patients with CKD should be monitored closely.

Interestingly, a recent clinical study involving 59 patients with COVID-19 found that 32 out of 51 patients (63%) had proteinuria, an indicator of impaired renal function [20]. With respect to other renal indicators, the authors also found that 19% and 27% of COVID-19 patients had elevated levels of plasma creatinine and urea nitrogen, respectively, and CT scans showed that 100% of 27 COVID-19 patients examined had renal abnormalities [20]. Importantly, a separate study of 52 COVID-19 patients (with 20 survivors and 32 nonsurviving patients) found that 15 patients (29%) presented with acute impaired renal function [9]. In addition, Zhou et al. reported that 15% of SARS-CoV-2-infected patients had AKI, compared to 50% in nonsurviving patients [12]. Similarly, Diao et al. reported that 27% of COVID-19 patients (23 out of 85) presented with AKI [21]. Overall, nearly 9.4% of critically ill patients admitted to the ICU with SARS-CoV-2 (55 out of 585 patients) had AKI; these results are summarized in Table 3.

Taken together, these findings indicate that kidney function should be closely monitored when treating patients with COVID-19, particularly patients with preexisting CKD and/or abnormal serum creatinine levels, blood urea nitrogen levels, or relevant CT findings [22]. Moreover, when treating COVID-19 patients with severe symptoms such as hyperkalemia, acidosis, and/or fluid overload in multiple organs, early continuous renal replacement therapy (CRRT) should be considered in order to maintain the patient's fluid balance, acid-base balance, and electrolyte balance. Importantly, CRRT may also be beneficial in alleviating cytokine storm and eliminating toxic metabolites in these patients.

### 2.3. Chronic Liver Disease Is Not Significantly Correlated with COVID-19 Severity, but Patients with Severe COVID-19 Are more Likely to Develop Acute Liver Dysfunction

**Figure 5:**
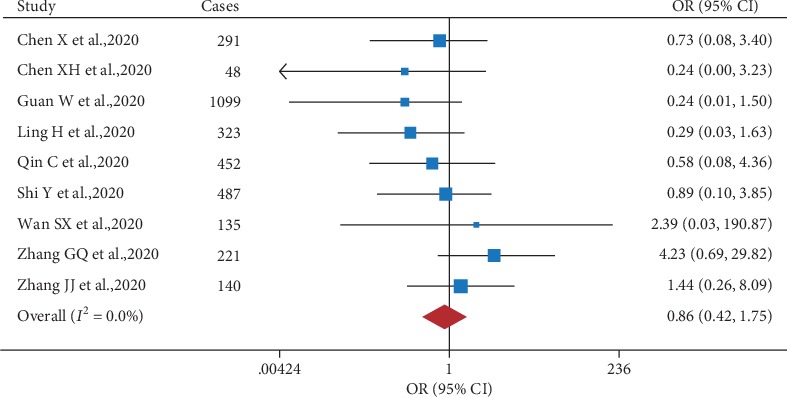
Forest plot showing the effect of comorbid chronic liver disease on the risk of severe COVID-19 in SARS-CoV-2-infected patients.

To our surprise, our analysis revealed that unlike cardiovascular disease and kidney disease, preexisting chronic liver disease (CLD) was not significantly correlated with COVID-19 severity. This conclusion was based on three separate lines of evidence. First, our meta-analysis revealed no significant correlation between CLD and severe COVID-19, with an overall OR of 0.86 (95% CI: 0.42, 1.75) and low heterogeneity (*I*^2^ = 0.0%) (Figure 5). Second, we found that the majority of COVID-19 patients with CLD did not require admittance to the ICU, suggesting a less severe disease course in this subset of patients. Consistent with this finding, Chen et al. reported that 13 out of 15 patients (86.7%) with both COVID-19 and CLD did not have severe disease [3]. Third, we found that many clinical reports regarding COVID-19 provided little or no information with respect to CLD. For example, in a large cohort study involving 1,099 patients with COVID-19, only 23 patients (2.1%) had hepatitis B [13]. Nevertheless, given the small number of patients with preexisting CLD analyzed, the effect of CLD on COVID-19 severity requires further study.

Next, we examined whether acute liver injury (ALI) plays a role in disease severity in SARS-CoV-2-infected patients, given the previous report that nearly 80% of SARS-CoV-infected patients (34 out of 43 patients) had abnormal liver function based on elevated serum ALT and/or AST levels [23] and given that serum ALT, AST, and LDH levels were higher in nonsurviving SARS patients compared to survivors [24]. Moreover, given the genetic and clinical similarities between the novel SARS-CoV-2 virus and the original SARS-CoV virus [5, 25], it is reasonable to speculate that ALI may also affect the severity of COVID-19. Indeed, Yao et al. reported that the incidence of ALI among severe COVID-19 patients is considerably higher compared to patients with moderate COVID-19 (77.3% vs. 27.8%, respectively) [26]. As summarized in Table 4, both AST and ALT levels were significantly higher in patients who had severe COVID-19 and/or were admitted to the ICU compared to patients who had moderate COVID-19 and were not admitted to the ICU. Strikingly, Guan et al. [13] examined a large set of laboratory data and found increased AST and increased ALT levels in 39.4% and 28.1%, respectively, of patients with severe COVID-19, compared to 18.2% and 19.8%, respectively, of patients with nonsevere COVID-19. However, it remains inconclusive with respect to whether ALI affects the mortality of COVID-19 patients. Yang et al. reported that the rate of liver dysfunction among 32 nonsurvivors and 20 survivors with severe COVID-19 disease was comparable, 28% and 30%, respectively [9]. Whereas in Cao et al.'s study, there was a significant difference (*P* < 0.001) in the rate of ALI, 76.5% (13 out of 17) in nonsurvivors and 24.7% (21 out of 85) in survivors [27]. Given the small number of patients in both studies, the effect of ALI on COVID-19 mortality requires further study.

### 2.4. Preexisting Diabetes Is a Predictive Factor for Severe COVID-19

**Figure 6:**
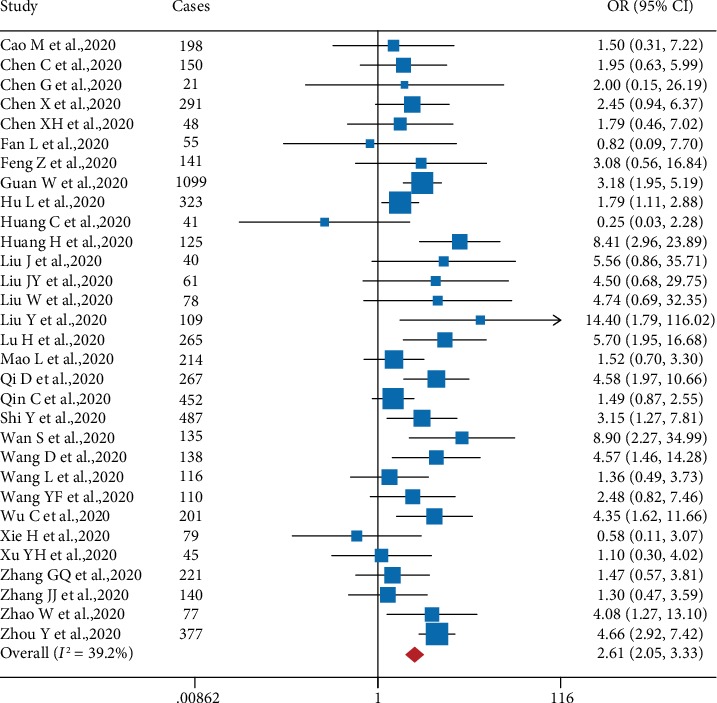
Forest plot showing the effect of comorbid diabetes on the risk of severe COVID-19 in SARS-CoV-2-infected patients.

Diabetes is a known risk factor for poorer outcome in patients who develop respiratory disease [28]; however, the association between diabetes and COVID-19 severity has not been examined systematically. We therefore performed a meta-analysis in order to examine the putative association between preexisting diabetes and COVID-19 severity. As shown in Figure 6, our analysis revealed that patients who present with diabetes have a significantly increased risk (OR: 2.61; 95% CI: 2.05, 3.33) of developing severe COVID-19 compared to nondiabetic patients, with moderate study heterogeneity (*I*^2^ = 39.2%). This finding is consistent with a previous retrospective study by Yang et al. showing that both preexisting diabetes (OR: 3.0; 95% CI: 1.4, 6.3) and preexisting hyperglycemia (OR: 3.3; 95% CI: 1.4, 7.7) were independent predictors of SARS-related death [29]. The same authors also found that during the course of a SARS infection, the patients' fasting plasma glucose levels were inversely correlated with arterial oxygenation (SaO_2_) and directly correlated with mortality and hypoxia [29]. Moreover, a meta-analysis of Middle East respiratory syndrome (MERS) studies by Badawi and Ryoo revealed that 51% (95% CI: 36%, 66%) of severe MERS-CoV-infected patients had diabetes [30], and a meta-analysis by Matsuyama et al. found that preexisting diabetes was associated with an increased risk of developing severe MERS-CoV-related complications (OR: 1.8; 95% CI: 1.5, 2.1) [31].

### 2.5. Publication Bias and Sensitivity Analysis

Next, we examined publication bias by generating funnel plots (Supplemental Figures 1-5), which revealed no evidence of publication bias for hypertension, CVD, CKD, CLD, or diabetes. In addition, both Egger's linear regression test and Begg's rank correlation test also showed no significant publication bias for each comparison (Supplemental Table 1). In addition, a sensitivity analysis revealed that no single study affected the pooled results or total effect size (Supplemental Figures 6-10).

## 3. Discussion

COVID-19 patients can present with a wide range of symptoms [6]. Although the majority of SARS-CoV-2-infected patients have relatively mild symptoms, a considerable number of patients develop severe disease.

The presence of a preexisting chronic disease has been suggested as a possible risk factor for increased disease severity in SARS patients [32, 33]. Consistent with previous reports, we found that preexisting hypertension, CVD, CKD, and diabetes are strongly associated with increased disease severity and poor prognosis in COVID-19 patients. To our surprise, we found no correlation between CLD and COVID-19 severity; this finding may be due to a sparing of viral attack in hepatocytes [34] and/or the liver's strong tolerance and ability to regenerate [35]. In addition, most CLD patients have virus-induced hepatitis that is typically treated with anti-inflammatory and/or antiviral drugs, which may partially mitigate the severity of COVID-19 upon SARS-CoV-2 infection [36]. Importantly, we found that impaired organ function, including acute cardiac injury and acute kidney injury, is strongly correlated with increased mortality in COVID-19 patients.

### 3.1. Lessons Learned from SARS

Although the clinical characteristics and risk factors for developing severe COVID-19 are largely unknown, previous knowledge obtained from studying SARS may provide valuable insights.

Recent evidence suggests that the novel SARS-CoV-2 virus and the original SARS-CoV virus use the same cell entry receptor, the ACE2 protein [37], which is expressed at high levels on the surface of pulmonary epithelial cells, myocardial cells, and arterial smooth muscle cells [38]. Ding et al. [39] systematically examined the presence of SARS-CoV in tissues of deceased SARS patients using immunohistochemistry and *in situ* hybridization; the authors found that SARS-CoV was present in the lungs, small intestine, kidneys, liver, pancreas, cerebrum, and other tissues, indicating that ACE2-expressing organs may serve as direct targets of SARS-CoV. Furthermore, SARS-CoV uses the ACE2 protein for cellular entry [40–42] and uses the cellular serine protease TMPRSS2 for viral spike protein priming [43–45]. A recent study confirmed that the closely related SARS-CoV-2 also uses both ACE2 and TMPRSS2 [46]. It is reported that the coding region variants and eQTL variants for ACE2 might also contribute to differential susceptibility or response to SARS-CoV-2 [47]. In addition, other proteins such as CD147 may also be employed by both SARS-CoV [48] and SARS-CoV-2 [49] during virus transmission. Nevertheless, it is noted that a subset of previously healthy and even relatively young COVID-19 patients has been killed by SARS-CoV-2, suggesting that the patients' genomes for DNA variations might have an impact on the disease severity and mortality [50, 51].

Similar to observations in COVID-19 patients, SARS patients also develop cardiovascular complications, including impaired left ventricular function [52]. Strikingly, even 12 years after their SARS-CoV infection, half of all patients have residual cardiovascular abnormalities [53]. Moreover, Oudit et al. found that more than one-third of archived SARS-infected heart samples obtained postmortem had evidence of myocardial infection at the time of death [54].

With respect to kidney injury, Chu et al. found that 6.7% of patients (36 out of 536) with SARS developed AKI with a median interval of 20 days (range: 5-48 days) following the onset of viral infection [55]; strikingly, the vast majority of these 36 SARS patients with AKI (91.7%, or 33 patients) eventually died, compared to a mortality rate of only 8.8% among SARS patients without AKI [55]. These results reinforce the notion that AKI may serve as a major risk factor contributing to the increased mortality rate among SARS patients [55]. Accordingly, renal function should be monitored in COVID-19 patients, thus providing a possible prognostic indicator of poor outcome.

Consistent with our findings, impaired liver function has also been associated with SARS severity [24]. Tong et al. studied 91 nonsevere SARS cases and 23 severe SARS cases (including 11 deaths) and found liver dysfunction in 95.7% of patients with severe SARS compared to 68.1% of nonsevere cases [56].

### 3.2. Possible Mechanisms Underlying Comorbid Chronic Diseases, Organ Injuries, and COVID-19 Severity

Currently, the mechanism underlying the development of ACI in SARS-CoV-2-infected patients is poorly understood. However, an important component of the renin-angiotensin system, ACE2 (angiotensin-converting enzyme 2), is a membrane-anchored carboxypeptidase that converts angiotensin II into angiotensin 1-7, thereby reducing the molecular and cellular effects of angiotensin II [57]. Importantly, ACE2 is expressed throughout the lungs but is also expressed in the cardiovascular system, where it has direct effects on cardiac function [58]. In support of this enzyme's important role in cardiac function, loss of ACE2 in mice causes severely impaired cardiac contractility and increases susceptibility to experimentally induced heart failure [59, 60].

One possible mechanism underlying the development of ACI during COVID-19 treatment may be drug-induced cardiotoxicity. For example, although chloroquine appears to block SARS-CoV-2 infection *in vitro* and has been recommended for clinical use by the National Health Commission of China [61], both chloroquine and its derivative hydroxychloroquine have been reported to cause cardiac side effects, including impaired conduction and hypertrophic cardiomyopathy [62]. Other drugs recommended for treating COVID-19, including interferon alpha and ribavirin, may also potentially cause cardiac damage [63]. Moreover, virus-induced cytokine storm and pneumonia-associated hypoxia may also contribute to the development ACI and/or the progression of ACI into heart failure in critically ill SARS-CoV-2-infected patients [64].

According to the recent study, immunohistochemistry showed that SARS-CoV-2 NP antigen was accumulated in kidney tubules, with severe acute tubular necrosis but without evidence of glomerular pathology or tubulointerstitial lymphocyte infiltration [21]. It is reasonable to speculate that the molecular interaction between the SARS-CoV-2 virus and the ACE2 enzyme in the kidneys of COVID-19 patients might play a role. In addition, immune-mediated kidney injury may also play a role. Indeed, a growing number of clinical studies have shown that the levels of various cytokines and chemokines—including IL-2, IL-7, IL-10, granulocyte-colony stimulating factor (GCSF), IP-10, monocyte chemoattractant protein-1 (MCP1), macrophage inflammatory protein-1*α* (MIP1A), and TNF-*α*—were significantly higher in severe COVID-19 patients compared to nonsevere patients [5]. Recently, Xu et al. reported increased numbers of peripheral CCR4^+^CCR6^+^ Th17 cells in a 50-year-old male patient with COVID-19 [65], suggesting the possible presence of SARS-CoV-2-induced inflammatory damage to the patient's tissues.

As with acute cardiac injury, drug-related toxicity may also explain the increased incidence of acquired AKI in SARS-CoV-2-infected patients. According to the National Health Commission of China's Diagnosis and Treatment of New Coronavirus Pneumonia guidelines [22], glucocorticoids, lopinavir/ritonavir, and ribavirin are treatment options for COVID-19. Interestingly, glucocorticoids provide a renoprotective effect in AKI via glucocorticoid-induced leucine zipper- (GILZ-) induced immunosuppression [66]. In contrast, lopinavir/ritonavir is widely used to treat AIDS and has been reported to cause renal tubular dysfunction and CKD [67]. Finally, ribavirin has been associated with a poor viral response and an increased prevalence of side effects in patients with low estimated glomerular filtration rate (eGFR) [68].

Although the underlying mechanism remains unclear, several factors may contribute to this putative association between ALI and severe COVID-19. First, the SARS-CoV-2 virus may directly cause liver damage. Chai et al. reported that cholangiocytes—but not hepatocytes—express ACE2, supporting the notion of virus-induced liver damage via ACE2-expressing cholangiocytes [69]. Further support comes from a report by Zhang et al. that 54% of COVID-19 patients had increased levels of gamma-glutamyl transferase, a diagnostic biomarker for cholangiocyte damage [70]. A second possible mechanism is that ALI in COVID-19 patients may result from a dysregulated inflammatory response, possibly including excessive activation of immune cells and subsequent inflammatory cytokine storm [71]. Finally, liver toxicity due to drugs used to treat COVID-19, including acetaminophen-containing antipyretics and/or lopinavir/ritonavir, may cause acute liver toxicity [72].

As for diabetes, although the mechanism underlying the relationship between diabetes and the severity of coronavirus-related disease is currently unknown, a study by Yang et al. [34] showed that ACE2 may be robustly expressed in pancreatic islet cells, suggesting that these cells could be targeted by both SARS-CoV and SARS-CoV-2. Moreover, *Ace2* knockout mice have impaired pancreatic *β*-cell function [73], indicating a possible correlation between SARS-CoV-2 infection and diabetes. Given the result of our meta-analysis, we recommend that COVID-19 patients with preexisting diabetes should be managed closely in order to prevent severe disease symptoms.

### 3.3. Conclusion and Outlook

**Figure 7:**
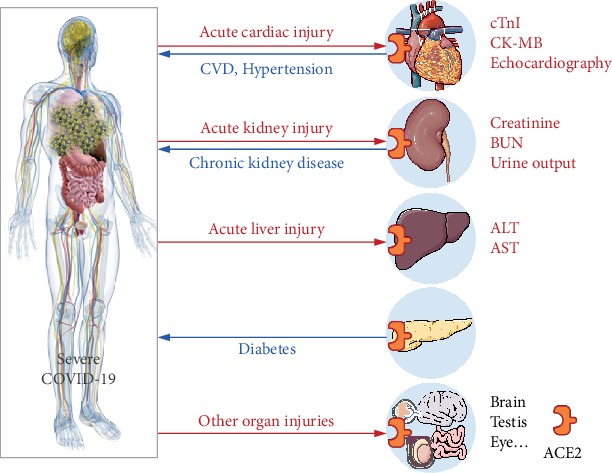
Schematic diagram depicting the putative association between severe COVID-19 and the indicated preexisting chronic diseases and affected organs. The blue line indicates the association between preexisting chronic diseases and COVID-19 severity. The red line indicates organ injuries observed in COVID-19 patients. Expression of ACE2 in the indicated organs is indicated. ACE2: angiotensin-converting enzyme 2; ALT: alanine transaminase; AST: aspartate aminotransferase; BUN: blood urea nitrogen; CK-MB: creatine kinase-MB; cTnI: cardiac troponin I; CVD: cardiovascular disease.

Our systematic review and meta-analysis support the notion of a strong correlation between COVID-19 severity and hypertension, CVD, CKD, and diabetes, four chronic diseases that are relatively common in the general population. An overview of the factors associated with severe COVID-19 is shown in Figure 7, summarizing the strong correlation with comorbidities and various forms of organ injury. Although the full clinical spectrum of COVID-19 severity is not currently known, several factors may contribute to the elevated risk associated with impaired organ function. First, SARS-CoV-2 can attack the wide range of organs and tissues that express the receptor protein ACE2 [34, 38]. Second, several chronic comorbidities, including hypertension, CVD, CKD, and diabetes, may render the affected organs and tissues susceptible to virus infection via an impaired immune response [74]. Third, certain antiviral drugs such as chloroquine [62], ribavirin [63, 68], and lopinavir/ritonavir [67] have side effects that can include organ damage. Fourth, acute respiratory distress syndrome- (ARDS-) induced hypoxia can promote damage in organs outside of the respiratory system [64, 75]. Finally, secondary infection by other pathogens may contribute to acute organ damage [5].

Despite the large sample size (6,263 COVID-19 cases from 34 clinical studies included) and the up-to-date overview of COVID-19, this study has several limitations. Firstly, studies included in this study primarily used retrospective cohorts, which are limited in their ability to infer definitive causality. Most recently, scientists and clinicians across the globe have responded to the ongoing coronavirus pandemic with a huge, high-quality global research effort to find a treatment for COVID-19. Secondly, nineteen out of thirty-four studies included in the meta-analysis were from preprint manuscripts, which are not peer reviewed. Thirdly, to ensure feasibility of this study, eligibility criteria were that data on COVID-19 patients were available in the published reports of the studies. It is noted that all included original clinical cohort studies were performed in China. More studies with broad geographic areas are likely to evolve over time, which may help to cross-validate the findings.

Previous meta-analyses reported hypertension [76–78] and CVD [76, 77] were correlated with COVID-19 severity. Our results support this notion with more clinical evidence. Notably, our headline findings are preexisting chronic kidney disease and diabetes have strong associations with increased COVID-19 severity, whereas chronic liver disease showed no correlation with the disease severity of COVID-19.

In summary, given the high risk of severe disease and the high mortality rate among SARS-CoV-2-infected patients who present with a chronic disease, and given that impaired organ function is correlated with high mortality rates, treating physicians and other healthcare providers should closely monitor and manage these vulnerable patients, particularly COVID-19 patients who develop severe disease and/or are admitted to the ICU.

## 4. Materials and Methods

### 4.1. Search Strategy

The databases PubMed, Embase, Web of Science, medRxiv, and bioRxiv were searched for all articles published through April 6, 2020, with no language restrictions, using the following keywords: “2019-nCoV” OR “SARS-CoV-2” OR “COVID-19” OR “new coronary pneumonia” OR “corona virus” OR “novel coronavirus” OR “nCoV”.

### 4.2. Study Selection

This systematic review and meta-analysis was conducted according to the PRISMA guidelines. Studies that satisfied the following three criteria were included in our meta-analysis: (1) the study was a clinical observation in humans; (2) the study included COVID-19 patient information; and (3) the study included information regarding comorbidity and/or organ injury. In addition, we excluded case studies involving only one COVID-19 patient and studies that were published as a narrative review, comment, opinion piece, methodological report, editorial, letter, or conference abstract.

### 4.3. Data Extraction

Data were extracted using a standardized data collection form. Detailed information was extracted from each included article, including the first author, publication date, study location, study design, patients' gender and age, sample size, comorbidity and organ injury, COVID-19 severity, and mortality.

### 4.4. Statistical Analysis and Data Synthesis

Meta-analyses were conducted in order to evaluate the association between various factors and the risk of developing severe COVID-19 [79]. The pooled results for use in the forest plots were analyzed using a random-effects model. Heterogeneity among the studies was estimated using the *I*^2^ statistic, with values of 0-25%, 25.1-75%, and 75.1-100% representing a low, moderate, and high degree of heterogeneity, respectively.

Publication bias was evaluated using contour-enhanced funnel plots, Egger's linear regression test, and Begg's rank correlation test, with significance set to *P* < 0.10. A sensitivity analysis was performed in order to examine the effect of individual studies by omitting 1 study at a time [80]. All statistical analyses were performed using Stata statistical software version 12 (StataCorp), and all *P* values were 2-sided with a significance level of 0.05 except where noted otherwise.

## Figures and Tables

**Table 1:** Characteristics of the 34 studies included in the meta-analysis.

First author, source, year	Patient geographic location	Total cases	Age in years, mean ± SD or median (range)	COVID-19 severity		Extracted disease comorbidity
				ICU and/or severe/ARDS COVID-19, *n* (%)	Non-ICU and/or nonsevere COVID-19, *n* (%)	
Cao M, medRxiv, 2020 [2]	Shanghai	198	50.1 ± 16.3	19 (9.6%)	179 (90.4%)	Hypertension, CVD, diabetes
Chen C, Zhonghua Xin Xue Guan Bing Za Zhi, 2020 [81]	Wuhan	150	59 ± 16	24 (16.0%)	126 (84.0%)	Hypertension, CVD, diabetes
Chen G, J Clin Invest, 2020 [82]	Wuhan	21	56 (50-65)	11 (52.4%)	10 (47.6%)	Hypertension, diabetes
Chen X, medRxiv, 2020 [3]	Changsha	291	46 (34-59)	50 (17.2%)	241 (82.8%)	Hypertension, CVD, CKD, CLD, diabetes
Chen XH, medRxiv, 2020 [4]	Wuhan	48	64.6 ± 18.1	27 (56.3%)	21 (43.7%)	Hypertension, CVD, CLD, diabetes
Fan L, medRxiv, 2020 [83]	Shenyang	55	46.8	8 (14.5%)	47 (85.5%)	CVD, diabetes
Feng Z, medRxiv, 2020 [84]	Changsha	141	44 (34-55)	15 (10.6%)	126 (89.4%)	Hypertension, CVD, diabetes
Guan W, N Engl J Med, 2020 [13]	National	1,099	47 (35-58)	173 (16.0%)	926 (84.0%)	Hypertension, CVD, CKD, CLD, diabetes
Hu L, medRxiv, 2020 [85]	Wuhan	323	61 (23-91)	172 (53.3%)	151 (46.7%)	Hypertension, CVD, CKD, CLD, diabetes
Huang C, Lancet, 2020 [5]	Wuhan	41	49 (41-58)	13 (32.0%)	28 (68.0%)	Hypertension, CVD, diabetes
Huang H, medRxiv, 2020 [86]	Guangzhou	125	44.87 ± 18.55	32 (25.6%)	93 (74.4%)	Hypertension, diabetes
Li KH, Invest Radiol, 2020 [87]	Chongqing	83	45.5 ± 12.3	25 (30.1%)	58 (69.9%)	Hypertension
Li Y, Curr Med Sci, 2020 [88]	Wuhan	25	NA	9 (36.0%)	16 (64.0%)	Hypertension, CVD
Liu J, medRxiv, 2020 [15]	Wuhan	40	48.7 ± 13.9	13 (33.0%)	27 (68.0%)	Hypertension, diabetes
Liu JY, medRxiv, 2020 [89]	Beijing	61	40 (1-86)	17 (28.0%)	44 (72.0%)	Hypertension, diabetes
Liu L, medRxiv, 2020 [90]	Chongqing	51	45 (34-51)	7 (13.7%)	44 (86.3%)	Hypertension
Liu W, Chin med J (Engl), 2020 [91]	Wuhan	78	38 (33-57)	11 (14.1%)	67 (85.9%)	Hypertension, diabetes
Liu Y, medRxiv, 2020 [92]	Wuhan	109	55 (43-66)	53 (48.6%)	56 (51.4%)	Hypertension, CVD, CKD, diabetes
Lu H, medRxiv, 2020 [16]	Shanghai	265	NA	22 (8.3%)	243 (91.7%)	Hypertension, CVD, CKD, diabetes
Mao L, medRxiv, 2020 [93]	Wuhan	214	52.7 ± 15.5	88 (41.1%)	126 (58.9%)	Hypertension, CKD, diabetes
Qi D, medRxiv, 2020 [94]	Chongqing	267	48 (20-80)	50 (18.7%)	217 (81.3%)	Hypertension, diabetes
Qin C, Clin Infect Dis, 2020 [95]	Wuhan	452	58 (47-67)	286 (63.3%)	166 (36.7%)	Hypertension, CVD, CKD, CLD, diabetes
Shi Y, Crit Care, 2020 [96]	Zhejiang	487	46 ± 19	49 (10.1%)	438 (89.9%)	Hypertension, CVD, CKD, CLD, diabetes
Wan S, J Med Viro, 2020 [97]	Chongqing	135	47 (36-55)	40 (29.6%)	95 (70.4%)	Hypertension, CVD, CLD, diabetes
Wang D, JAMA, 2020 [6]	Wuhan	138	56 (42-68)	36 (26.0%)	102 (74.0%)	Hypertension, CVD, CKD, diabetes
Wang L, Am J Nephrol, 2020 [98]	Wuhan	116	54 (38-69)	57 (49.1%)	59 (50.9%)	Hypertension, CKD, diabetes
Wang YF, medRxiv, 2020 [99]	Wuhan	110	NA	38 (34.5%)	72 (65.5%)	Hypertension, diabetes
Wu C, JAMA Intern Med, 2020 [100]	Wuhan	201	51 (43-60)	84 (41.8%)	117 (58.2%)	Hypertension, CVD, diabetes
Xie H, Liver Int, 2020 [101]	Wuhan	79	60 (48-66)	28 (35.4%)	51 (64.6%)	Hypertension, CVD, diabetes
Xu YH, medRxiv, 2020 [11]	Guangzhou	45	56.7 ± 15.4	20 (44.4%)	25 (55.6%)	Hypertension, CVD, diabetes
Zhang GQ, medRxiv, 2020 [10]	Wuhan	221	55 (39-66.5)	55 (24.9%)	166 (75.1%)	Hypertension, CVD, CKD, CLD, diabetes
Zhang JJ, Allergy, 2020 [102]	Wuhan	140	57 (25-87)	58 (41.4%)	82 (58.6%)	Hypertension, CVD, CKD, CLD, diabetes
Zhao W, medRxiv, 2020 [103]	Beijing	77	52 ± 20	20 (26.0%)	57 (74.0%)	Hypertension, CVD, CKD, diabetes
Zhou Y, medRxiv, 2020 [104]	Wuhan	377	NA	117 (31.0%)	260 (69.0%)	Hypertension, CVD, diabetes

ARDS: acute respiratory distress syndrome; CKD: chronic kidney disease; CLD: chronic liver disease; CVD: cardiovascular disease; NA: not available.

**Table 2:** Epidemiological characteristics of cardiac injury in COVID-19 patients.

First author, source, year	Location	No. of patients	No. of severe patients (%)	No. of patients with ACI (%)	Note
Cao J, Clin Infect Dis, 2020 [27]	Wuhan	102	18 (17.6%)	15 (14.7%)	12 ACI cases from 17 nonsurvivors 3 ACI cases from 85 survivors
Huang C, Lancet, 2020 [5]	Wuhan	41	13 (31.7%)	5 (12.2%)	4 ACI cases from 13 ICU patients 1 ACI case from 28 ICU patients
Wang D, JAMA, 2020 [6]	Wuhan	138	36 (26.1%)	10 (7.3%)	8 ACI cases from 36 ICU patients 2 ACI cases from 102 non-ICU patients
Hu L, medRxiv, 2020 [85]	Wuhan	323	172 (53.3%)	24 (7.4%)	13 ACI cases from 26 critical patients 9 ACI cases from 146 severe patients 2 ACI case from 151 nonsevere patients
Hui H, medRxiv, 2020 [7]	Beijing	41	7 (17.1%)	4 (9.8%)	3 ACI cases from 3 critical patients 1 ACI case from 4 severe patients
Shi S, JAMA Cardiol, 2020 [105]	Wuhan	416	NA	82 (19.7%)	42 deaths in 82 cases with ACI 15 deaths in 334 cases without ACI
Wan S, J Med Virol, 2020 [97]	Chongqing	135	40 (29.6%)	10 (7.4%)	2 ACI cases from 40 severe patients 8 ACI cases from 95 mild patients
Wu C, medRxiv, 2020 [8]	Wuhan	188	NA	21 (11.2%)	15 ICU cases and 6 deaths in the low TnI group (60 patients) 14 ICU cases and 6 deaths in the moderate TnI group (66 patients) 27 ICU cases and 31 deaths in the high TnI group (62 patients)
Xu YH, medRxiv, 2020 [11]	Guangdong	45	45 (100.0%)	10 (22.2%)	All 10 ACI cases from 20 patients required intubation
Yang X, Lancet Respir Med, 2020 [9]	Wuhan	52	52 (100.0%)	12 (23.1%)	9 ACI cases from 32 nonsurvivors 3 ACI cases from 20 survivors
Zhang GQ, medRxiv, 2020 [10]	Wuhan	221	55 (24.9%)	17 (7.7%)	16 ACI cases from 55 severe patients 1 ACI case from 166 nonsevere patients
Zhao W, medRxiv, 2020 [103]	Beijing	77	20 (26.0%)	2 (2.6%)	2 ACI cases from 20 severe patients
Zhou F, Lancet, 2020 [12]	Wuhan	191	119 (62.3%)	33 (17.3%)	32 ACI cases from 54 nonsurvivors 1 ACI case from 137 survivors

ACI: acute cardiac injury; TnI: troponin I.

**Table 3:** Epidemiological characteristics of kidney injury in COVID-19 patients.

First author, source, year	Location	No. of patients	No. of severe patients (%)	No. of patients with AKI (%)	No. of severe patients with AKI (%)
Guan W, N Engl J Med, 2020 [13]	China	1,099	173 (15.7%)	6 (0.6%)	5 (2.9%)
Hu L, medRxiv, 2020 [85]	Wuhan	323	152 (47.1%)	17 (5.3%)	15 (9.9%)
Huang C, Lancet, 2020 [5]	Wuhan	41	13 (31.7%)	3 (7.3%)	3 (23.1%)
Wan S, J of Med Viro, 2020 [97]	Chongqing	135	40 (29.6%)	5 (3.7%)	1 (2.5%)
Wang D, JAMA, 2020 [6]	Wuhan	138	36 (26.1%)	5 (3.6%)	3 (8.3%)
Xu YH, medRxiv, 2020 [11]	Guangdong	45	45 (100.0%)	7 (15.6%)	7 (15.6%)
Yang X, Lancet Respir Med, 2020 [9]	Wuhan	52	52 (100.0%)	15 (28.9%)	15 (28.9%)
Zhang GQ, medRxiv, 2020 [10]	Wuhan	221	55 (24.9%)	10 (4.5%)	8 (14.6%)
Zhao W, medRxiv, 2020 [103]	Beijing	77	20 (26.0%)	2 (2.6%)	1 (5.0%)

AKI: acute kidney injury.

**Table 4:** Epidemiological characteristics of liver injury in COVID-19 patients.

First author, source, year	Location	No. of patients	No. of severe patients (%)	Notes
Cao J, Clin Infect Dis, 2020 [27]	Wuhan	102	18 (17.6%)	13 cases of acute liver injury from 17 nonsurvivors, 21 cases of acute liver injury from 85 survivors.
Cao M, medRxiv, 2020 [2]	Shanghai	198	19 (9.6%)	Compared to non-ICU (moderate) patients, AST, ALT, and total bilirubin were significantly increased in ICU (severe) patients, while albumin was significantly decreased.
Chen G, J Clin Invest, 2020 [82]	Wuhan	21	11 (52.4%)	Compared to non-ICU (moderate) patients, AST, ALT, and LDH levels were significantly increased in ICU (severe) patients, while albumin was significantly decreased.
Fan L, medRxiv,2020 [83]	Shenyang	55	8 (14.5%)	11 cases of liver dysfunction from 47 mild patients, 6 cases of liver dysfunction from 8 severe patients.
Guan W, N Engl J Med, 2020 [13]	National	1,099	173 (15.7%)	Increased AST levels in 112 of 615 nonsevere patients, 56 of 142 severe patients, and increased ALT levels in 120 of 606 nonsevere patients, 38 of 135 severe patients.
Huang C, Lancet, 2020 [5]	Wuhan	41	13 (31.7%)	Elevated levels of AST were observed in 8 of 13 (61.5%) ICU patients and 7 of 28 (25%) non-ICU patients. Compared to non-ICU patients, ALT levels were significantly increased in ICU patients.
Huang H, medRxiv, 2020 [86]	Wuhan	125	32 (25.6%)	Compared to non-ICU (moderate) patients, AST and ALT levels were significantly increased in ICU (severe) patients.
Liu J, medRxiv, 2020 [15]	Wuhan	40	13 (32.5%)	Compared to non-ICU (moderate) patients, AST, ALT, and total bilirubin levels were significantly increased in ICU (severe) patients.
Lu H, medRxiv, 2020 [16]	Shanghai	265	22 (8.3%)	Compared to non-ICU (moderate) patients, AST, ALT, and LDH levels were significantly increased in ICU (severe) patients, while albumin was significantly decreased.
Wang D, JAMA, 2020 [6]	Wuhan	138	36 (26.1%)	Compared to non-ICU (moderate) patients, AST, ALT, prothrombin time, total bilirubin, and LDH were significantly increased in ICU (severe) patients.
Xu YH, medRxiv, 2020 [11]	Guangdong	45	45 (100.0%)	12 cases of liver dysfunction from 20 patients required intubation, 5 cases of liver dysfunction from 25 patients did not require intubation.
Yang X, Lancet Respir Med, 2020 [9]	Wuhan	52	52 (100.0%)	6 cases of liver dysfunction from 20 survivors, and 9 cases of liver dysfunction from 32 nonsurvivors.
Yao N, Zhonghua Gan Zang Bing Za Zhi, 2020 [26]	Shaanxi	40	17 (42.5%)	17 severe patients from 22 ALI cases, and 5 severe patients from 18 cases with normal liver dysfunction.
Zhang GQ, medRxiv, 2020 [10]	Wuhan	221	55 (24.9%)	Compared to non-ICU (moderate) patients, AST, ALT, prothrombin time, total bilirubin, and LDH were significantly increased in ICU (severe) patients.

ALI: acute liver injury; ALT: alanine transaminase; AST: aspartate aminotransferase; LDH: lactate dehydrogenase.

## Supplementary Materials

Supplementary Materials Supplemental Table 1: publication bias examined by Egger's linear regression test and Begg's rank correlation test. Supplemental Figure 1: funnel plots for hypertension analysis. Supplemental Figure 2: funnel plots for CVD analysis. Supplemental Figure 3: funnel plots for CKD analysis. Supplemental Figure 4: funnel plots for CLD analysis. Supplemental Figure 5: funnel plots for diabetes analysis. Supplemental Figure 6: sensitivity analysis for the association between hypertension and COVID-19 severity. Supplemental Figure 7: sensitivity analysis for the association between CVD and COVID-19 severity. Supplemental Figure 8: sensitivity analysis for the association between CKD and COVID-19 severity. Supplemental Figure 9: sensitivity analysis for the association between CLD and COVID-19 severity. Supplemental Figure 10: sensitivity analysis for the association between diabetes and COVID-19 severity.
