# Supplementary material for: Comorbid Chronic Diseases and Acute Organ Injuries Are Strongly Correlated with Disease Severity and Mortality among COVID-19 Patients: A Systemic Review and Meta-Analysis
Source: Research (Wash D C). 2020 Apr 19;2020:2402961. doi: 10.34133/2020/2402961 (PMC7187729; doi:10.34133/2020/2402961)
Supplement: Supplementary Materials — Supplemental Table 1: publication bias examined by Egger's linear regression test and Begg's rank correlation test. Supplemental Figure 1: funnel plots for hypertension analysis. Supplemental Figure 2: funnel plots for CVD analysis. Supplemental Figure 3: funnel plots for CKD analysis. Supplemental Figure 4: funnel plots for CLD analysis. Supplemental Figure 5: funnel plots for diabetes analysis. Supplemental Figure 6: sensitivity analysis for the association between hypertension and COVID-19 severity. Supplemental Figure 7: sensitivity analysis for the association between CVD and COVID-19 severity. Supplemental Figure 8: sensitivity analysis for the association between CKD and COVID-19 severity. Supplemental Figure 9: sensitivity analysis for the association between CLD and COVID-19 severity. Supplemental Figure 10: sensitivity analysis for the association between diabetes and COVID-19 severity. [file 2402961.f1.pdf]

Comorbid Chronic Diseases and Acute Organ Injuries Are Strongly Correlated with Disease Severity and Mortality among COVID-19 Patients: A Systemic Review and Meta-analysis

Xinhui Wang, Xuexian Fang, Zhaoxian Cai, Xiaotian Wu, Xiaotong Gao, Junxia Min, Fudi Wang.

The file includes:

SUPPLEMENTAL FIGURES 1-10

SUPPLEMENTAL TABLE 1

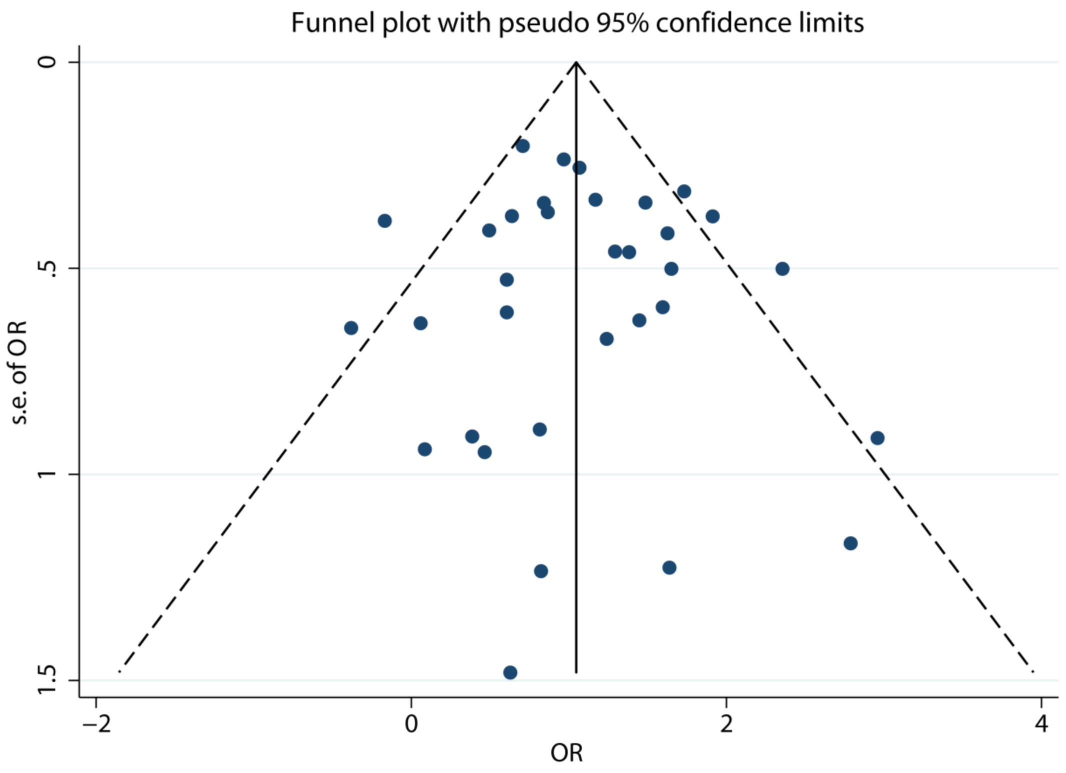

SUPPLEMENTAL FIGURE 1. Funnel plots for hypertension analysis. Funnel plots with pseudo 95% confidence limits were generated in order to identify possible publication bias of the included studies. The odd ratios are plotted against the standard error for the indicated hypertension indicators.

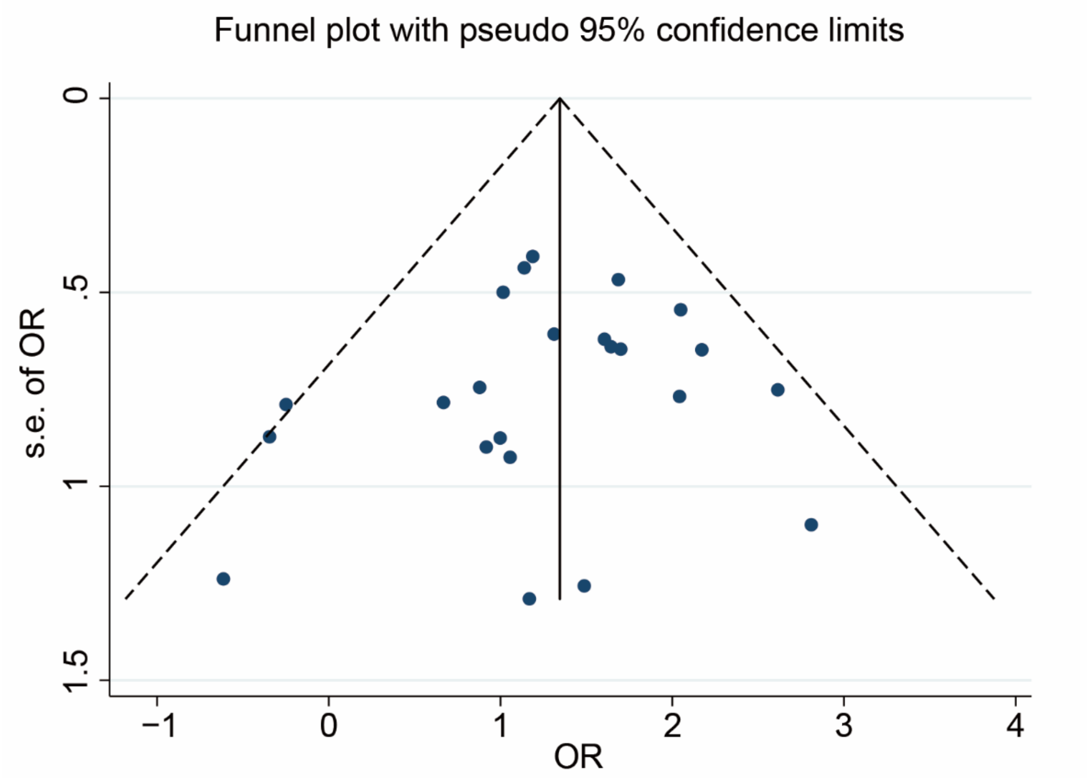

SUPPLEMENTAL FIGURE 2. Funnel plots for CVD analysis. Funnel plots with pseudo 95% confidence limits were generated in order to identify possible publication bias of the included studies. The odd ratios are plotted against the standard error for the indicated CVD indicators.

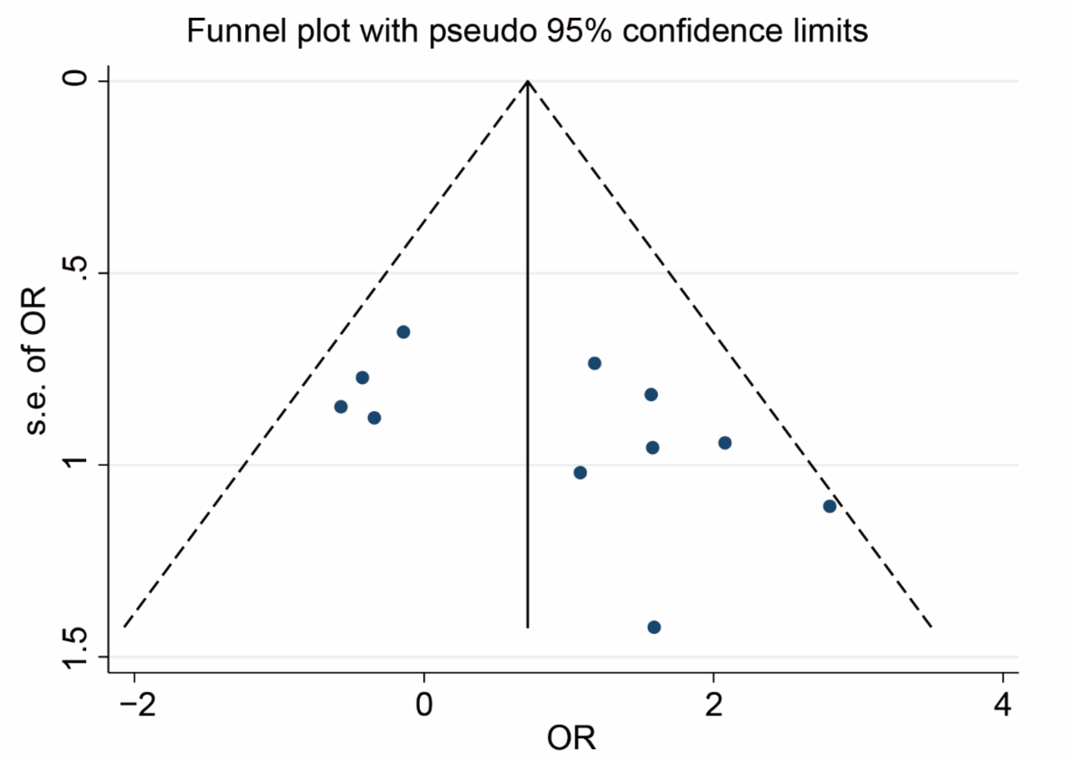

SUPPLEMENTAL FIGURE 3. Funnel plots for CKD analysis. Funnel plots with pseudo 95% confidence limits were generated in order to identify possible publication bias of the included studies. The odd ratios are plotted against the standard error for the indicated CKD indicators.

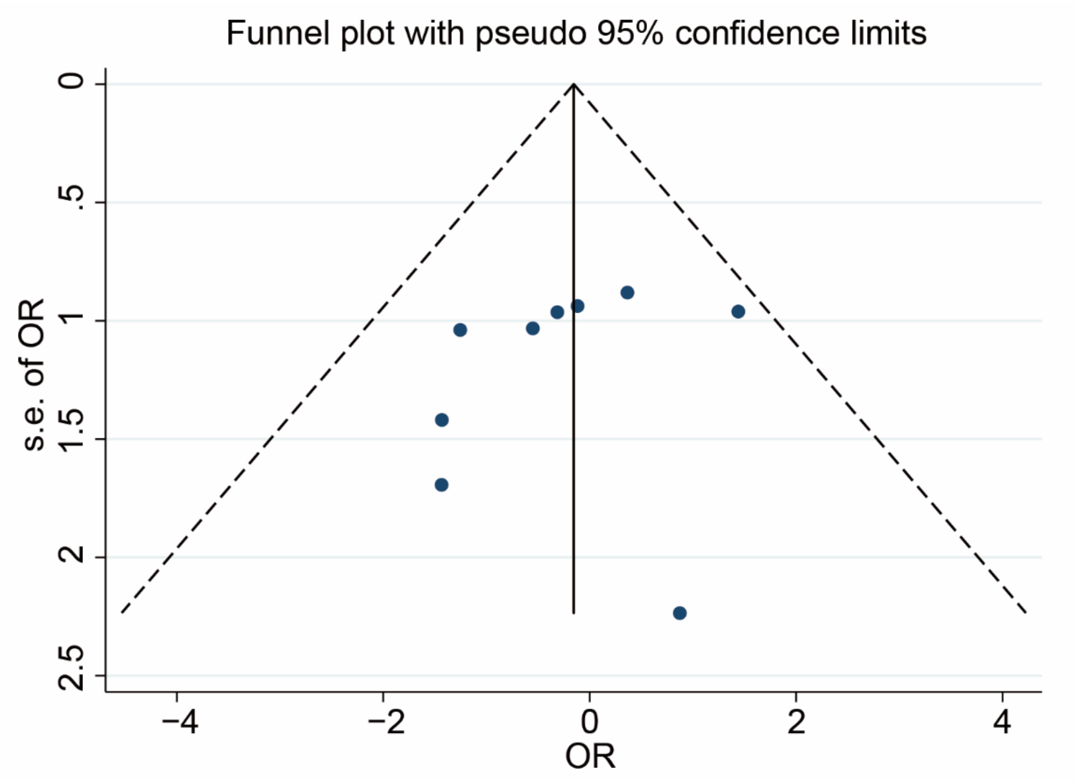

SUPPLEMENTAL FIGURE 4. Funnel plots for CLD analysis. Funnel plots with pseudo 95% confidence limits were generated in order to identify possible publication bias of the included studies. The odd ratios are plotted against the standard error for the indicated CLD indicators.

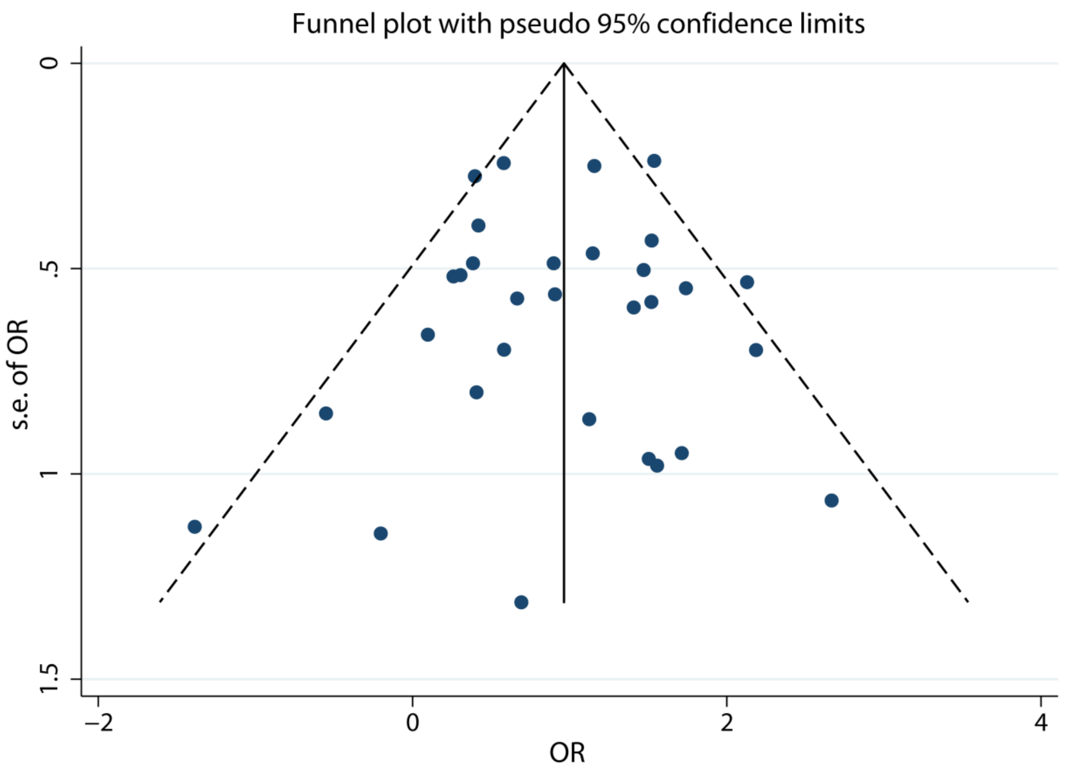

SUPPLEMENTAL FIGURE 5. Funnel plots for diabetes analysis. Funnel plots with pseudo 95% confidence limits were generated in order to identify possible publication bias of the included studies. The odd ratios are plotted against the standard error for the indicated glycemic indicators.

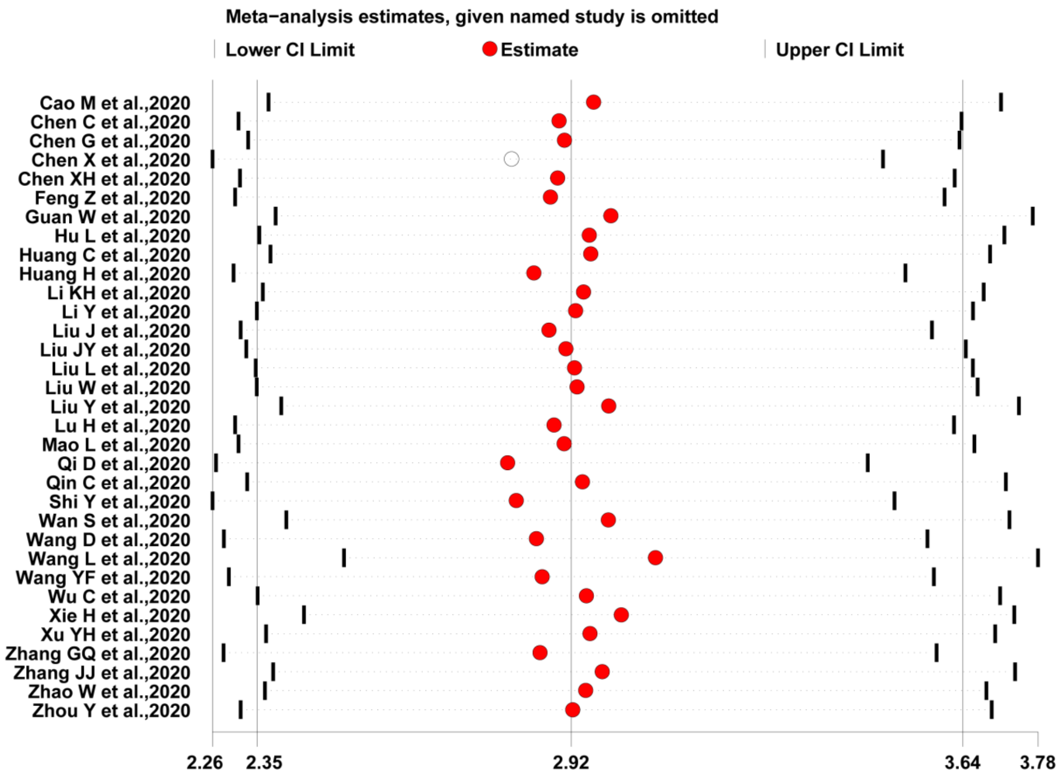

SUPPLEMENTAL FIGURE 6. Sensitivity analysis for the association between hypertension and COVID-19 severity. The results of a sensitivity test are shown for the indicated comorbidities. In each panel, each indicated study was omitted from the pooled analysis, and the effect on the total results was determined. Each circle and corresponding vertical tick represent the effect size and 95% CI after the corresponding study was omitted. For comparison, the three vertical lines indicate the positions of the effect size and the upper and lower limits of the 95% CI for the pooled results.

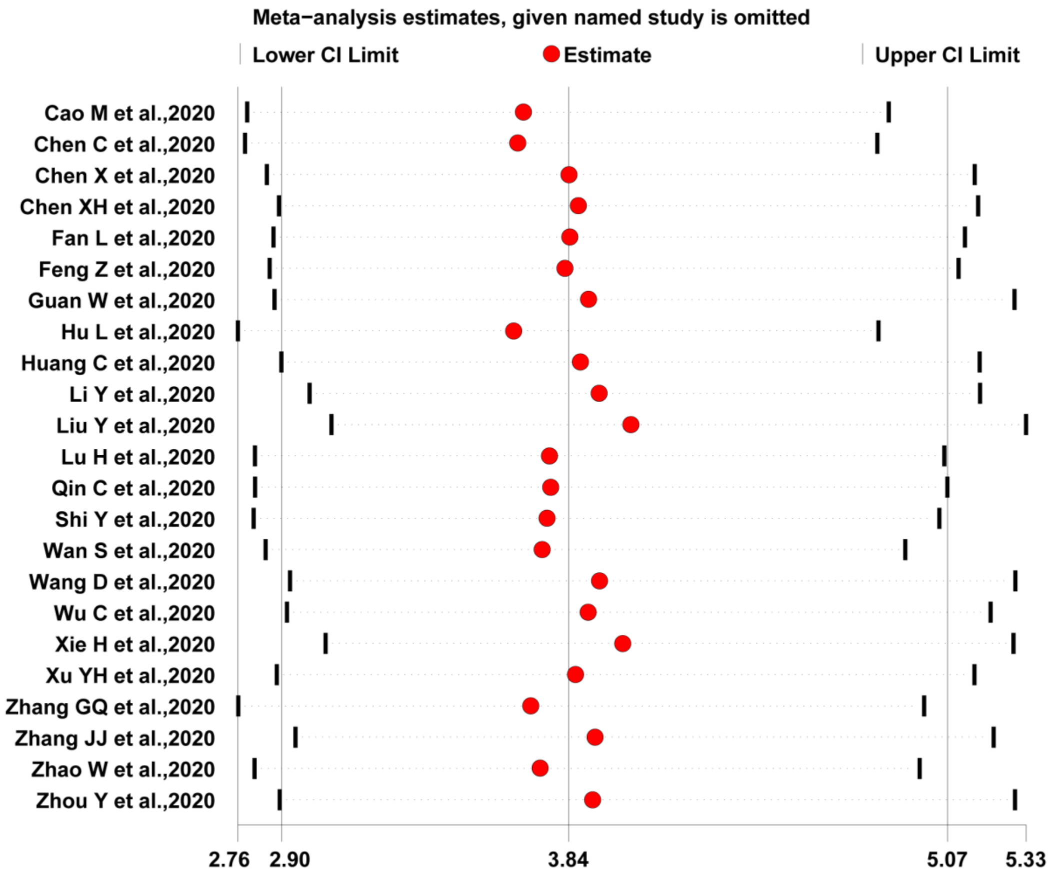

SUPPLEMENTAL FIGURE 7. Sensitivity analysis for the association between CVD and COVID-19 severity. The results of a sensitivity test are shown for the indicated comorbidities. In each panel, each indicated study was omitted from the pooled analysis, and the effect on the total results was determined. Each circle and corresponding vertical tick represent the effect size and 95% CI after the corresponding study was omitted. For comparison, the three vertical lines indicate the positions of the effect size and the upper and lower limits of the 95% CI for the pooled results.

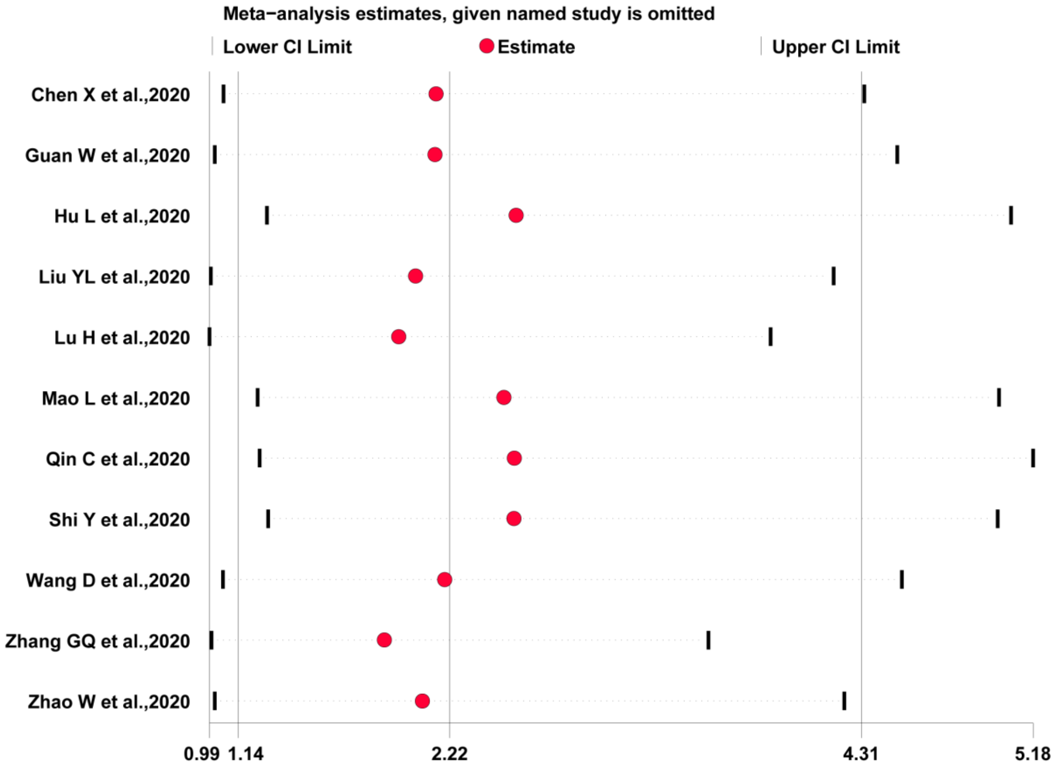

SUPPLEMENTAL FIGURE 8. Sensitivity analysis for the association between CKD and COVID-19 severity. The results of a sensitivity test are shown for the indicated comorbidities. In each panel, each indicated study was omitted from the pooled analysis, and the effect on the total results was determined. Each circle and corresponding vertical tick represent the effect size and 95% CI after the corresponding study was omitted. For comparison, the three vertical lines indicate the positions of the effect size and the upper and lower limits of the 95% CI for the pooled results.

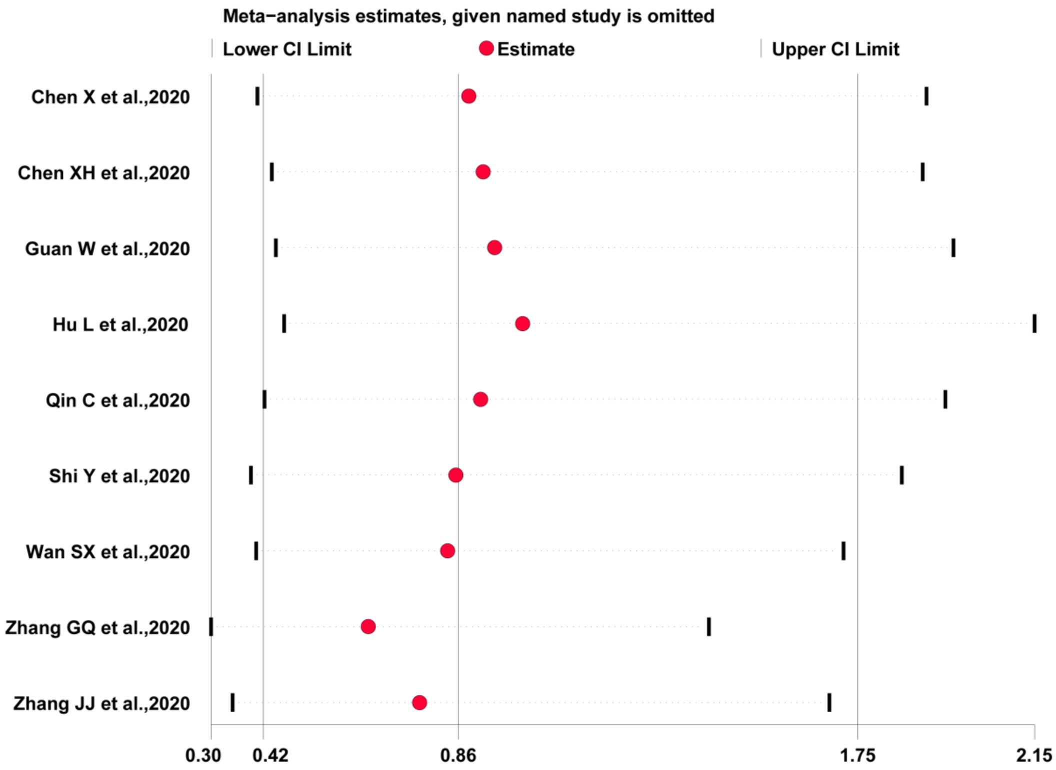

SUPPLEMENTAL FIGURE 9. Sensitivity analysis for the association between CLD and COVID-19 severity. The results of a sensitivity test are shown for the indicated comorbidities. In each panel, each indicated study was omitted from the pooled analysis, and the effect on the total results was determined. Each circle and corresponding vertical tick represent the effect size and 95% CI after the corresponding study was omitted. For comparison, the three vertical lines indicate the positions of the effect size and the upper and lower limits of the 95% CI for the pooled results.

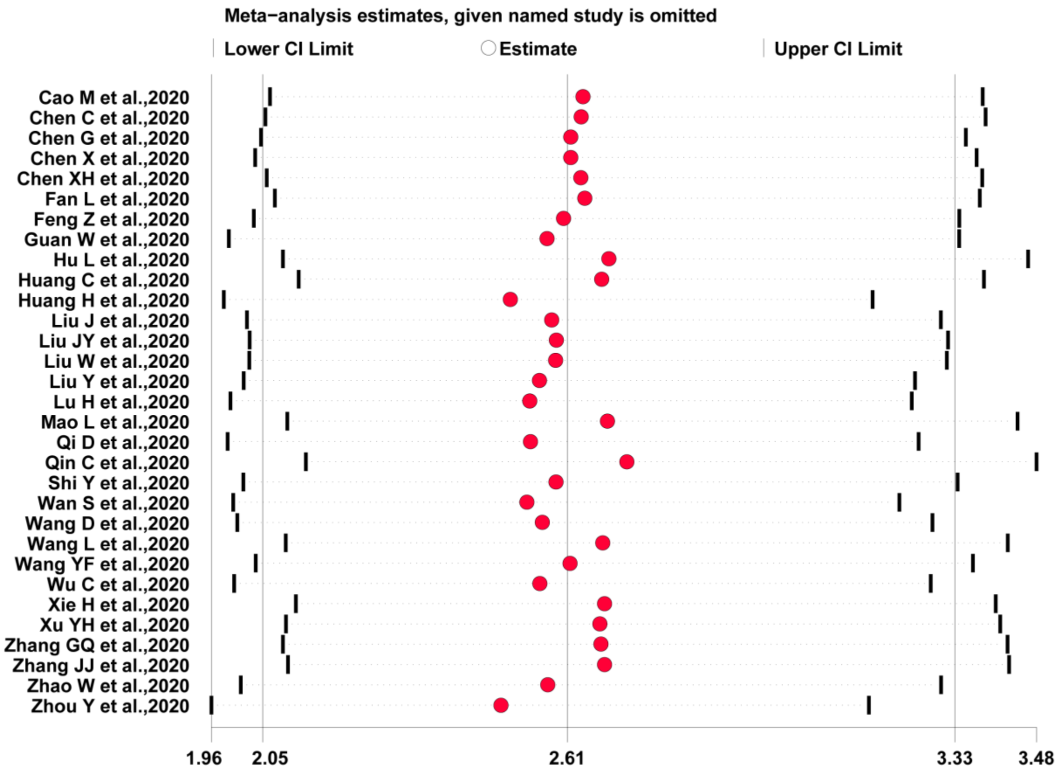

SUPPLEMENTAL FIGURE 10. Sensitivity analysis for the association between diabetes and COVID-19 severity. The results of a sensitivity test are shown for the indicated comorbidities. In each panel, each indicated study was omitted from the pooled analysis, and the effect on the total results was determined. Each circle and corresponding vertical tick represents the effect size and 95% CI after the corresponding study was omitted. For comparison, the three vertical lines indicate the positions of the effect size and the upper and lower limits of the 95% CI for the pooled results.

SUPPLEMENTAL TABLE 1. Publication bias examined by Egger's linear regression test and Begg's rank correlation test.

	***P* for Egger's test**	***P* for Begg's test**
**Hypertension**	0.67	0.96
**CVD**	0.45	0.71
**CKD**	0.19	0.21
**CLD**	0.46	0.18
**Diabetes**	0.87	0.95
